# Exploring the Value of Radiomics Features Based on B-Mode and Contrast-Enhanced Ultrasound in Discriminating the Nature of Thyroid Nodules

**DOI:** 10.3389/fonc.2021.738909

**Published:** 2021-10-14

**Authors:** Shi Yan Guo, Ping Zhou, Yan Zhang, Li Qing Jiang, Yong Feng Zhao

**Affiliations:** The Department of Ultrasound, Xiangya Third Hospital, Central South University, Changsha, China

**Keywords:** thyroid nodules, contrast-enhanced ultrasound, diagnosis performance, radiomics features, clinical value

## Abstract

**Background:**

With the improvement of ultrasound imaging resolution and the application of various new technologies, the detection rate of thyroid nodules has increased greatly in recent years. However, there are still challenges in accurately diagnosing the nature of thyroid nodules. This study aimed to evaluate the clinical application value of the radiomics features extracted from B-mode ultrasound (B-US) images combined with contrast-enhanced ultrasound (CEUS) images in the differentiation of benign and malignant thyroid nodules by comparing the diagnostic performance of four logistic models.

**Methods:**

We retrospectively collected and ultimately included B-US images and CEUS images of 123 nodules from 123 patients, and then extracted the corresponding radiomics features from these images respectively. Meanwhile, a senior radiologist combined the thyroid imaging reporting and data system (TI-RADS) and the enhancement pattern of the ultrasonography to make a graded diagnosis of the malignancy of these nodules. Next, based on these radiomics features and grades, logistic regression was used to help build the models (B-US radiomics model, CEUS radiomics model, B-US+CEUS radiomics model, and TI-RADS+CEUS model). Finally, the study assessed the diagnostic performance of these radiomics features with a comparison of the area under the curve (AUC) of the receiver operating characteristic curve of four logistic models for predicting the benignity or malignancy of thyroid nodules.

**Results:**

The AUC in the differential diagnosis of the nature of thyroid nodules was 0.791 for the B-US radiomics model, 0.766 for the CEUS radiomics model, 0.861 for the B-US+CEUS radiomics model, and 0.785 for the TI-RADS+CEUS model. Compared to the TI-RADS+CEUS model, there was no statistical significance observed in AUC between the B-US radiomics model, CEUS radiomics model, B-US+CEUS radiomics model, and TI-RADS+CEUS model (*P*>0.05). However, a significant difference was observed between the single B-US radiomics model or CEUS radiomics model and B-US+CEUS radiomics model (*P*<0.05).

**Conclusion:**

In our study, the B-US radiomics model, CEUS radiomics model, and B-US+CEUS radiomics model demonstrated similar performance with the TI-RADS+CEUS model of senior radiologists in diagnosing the benignity or malignancy of thyroid nodules, while the B-US+CEUS radiomics model showed better diagnostic performance than single B-US radiomics model or CEUS radiomics model. It was proved that B-US radiomics features and CEUS radiomics features are of high clinical value as the combination of the two had better diagnostic performance.

## Introduction

In recent years, thyroid nodules can be observed in up to 50 - 60% of healthy recipients, but only about 5% are proven to be malignant. As a result, when encountering patients with thyroid nodules today, clinicians are faced with the task of avoiding the overdiagnosis of low-risk cancers without jeopardizing the chances of identifying those rare late-stage or higher-risk tumors that will require timely and appropriate treatment (1, 2). Ultrasound as the principle means for the detection and risk stratification of thyroid nodules provides guidance for their biopsy and nonsurgical treatment (3). However, misdiagnosis and unnecessary operations are still prevalent for certain nodules because of interobserver and intraobserver variability, the overlap in morphological features of certain benign and malignant nodules, and different diagnostic criteria in different regions (4, 5). Fine-needle aspiration biopsy (FNAB) is currently the most reliable and cost-effective examination method for assessing thyroid nodules (6), which can effectively screen out benign thyroid nodules as well as reduce the rate of unnecessary surgeries (2). However, there are still 30-40% of FNAB procedures that generate nondiagnostic results due to various factors, such as the features of the nodules, and the experience of the physician and pathologist (7–9). In addition, it is not widely accepted as an invasive examination. Hence, researchers have been pursuing a precise, uniform, and non-invasive diagnostic method. The notion of radiomics was introduced by Lambin (10) in 2012. The visual image information can be transformed into deep-seated features for quantitative research. It gives us hope to achieve non-invasive precision medicine, which is widely used to evaluate tumors in all parts of the body. Based on a radiomics analysis method, we extracted the corresponding radiomics features from thyroid nodule ultrasound images to build logistic models, and evaluated the clinical application value of these features in differentiating benign and malignant thyroid nodules by comparing the diagnostic performance of the radiomics models with the model based on the grading of nodule malignancy by a senior radiologist.

## Study Subjects and Methods

### Study Subjects

This was a retrospective study; the subjects of our study were two-dimensional gray-scale images and contrast-enhanced ultrasound images of patients with thyroid nodules. All of the participants signed an informed consent form prior to the examination, with the approval of the hospital ethics committee. Inclusion criteria: Patients who had received a CEUS examination with complete imaging data in our hospital between September 2020 and August 2021, and had underwent thyroid fine-needle aspiration biopsy or pathological diagnoses within one week after the examination. Exclusion criteria: Patient nodules with an unclear pathological finding, and the proportion of cystic composition over 25% (11), in addition, we also discarded images with poor image quality.

### Image Collection

**Figure 1:**
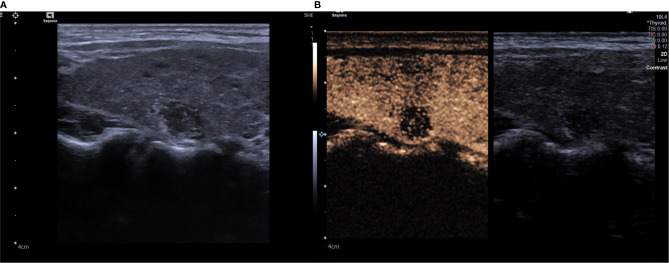
**(A)** A grayscale ultrasound image. We found a 9 x9mm solid hypoechoic nodule in the right lobe of a patient’s thyroid, this nodule has faint borders, irregular margin and microcalcifications inside. **(B)** A contrast ultrasound image, the nodule shown has low enhancement. The degree of malignancy risk of the nodule is classified as grade 5.

The Siemens X150 Vivi7 color doppler ultrasound diagnostic system was used to perform diagnoses, which was equipped with a 10L4 transducer for B-US and CEUS examination. Each patient lay on the inspection bed in the supine position and was told to fully extend their neck and breathe calmly. By carefully scanning every section of the thyroid nodule, we observed and recorded internal composition, internal echo, the boundary, the margin, aspect ratio, and calcification. Based on a previous study (12), solid nodules with an ill-defined border, irregular margin, hypoechogenicity, an aspect ratio >1, and microcalcification were found to be significantly related to malignancy. Then, the maximum long-axis section of the thyroid nodules was preserved. Next, we adjusted the focus to the lower edge of the thyroid nodules, switching to contrast-enhanced ultrasound mode. This study was conducted using the SonoVue contrast agent (Bracco, Milan, Italy). We continuously monitored the dynamic perfusion process of the nodules in real-time after quickly pushing the contrast agent into the peripheral vein, we then exported the B-US images and CEUS clips in the dicom format (**Figure 1**). These examinations and evaluations were performed by a senior radiologist with over 20 years of experience in diagnostic ultrasound of thyroid disease.

### Processing Images

**Figure 2:**
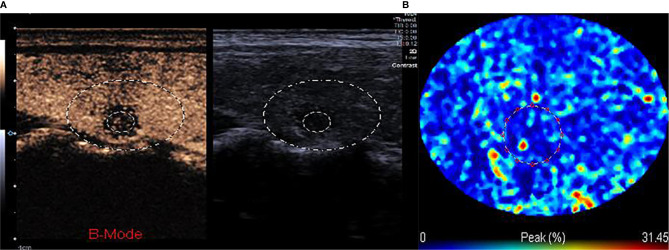
**(A, B)** The TIC analysis curve of the CEUS image. First, we selected the target area with a large circle, second, we drew the ROI inside the nodule with a small circle. According to the TIC curve in the small circle, we selected the frame corresponding to the moment when internal nodule perfusion reached its peak.

**Figure 3:**
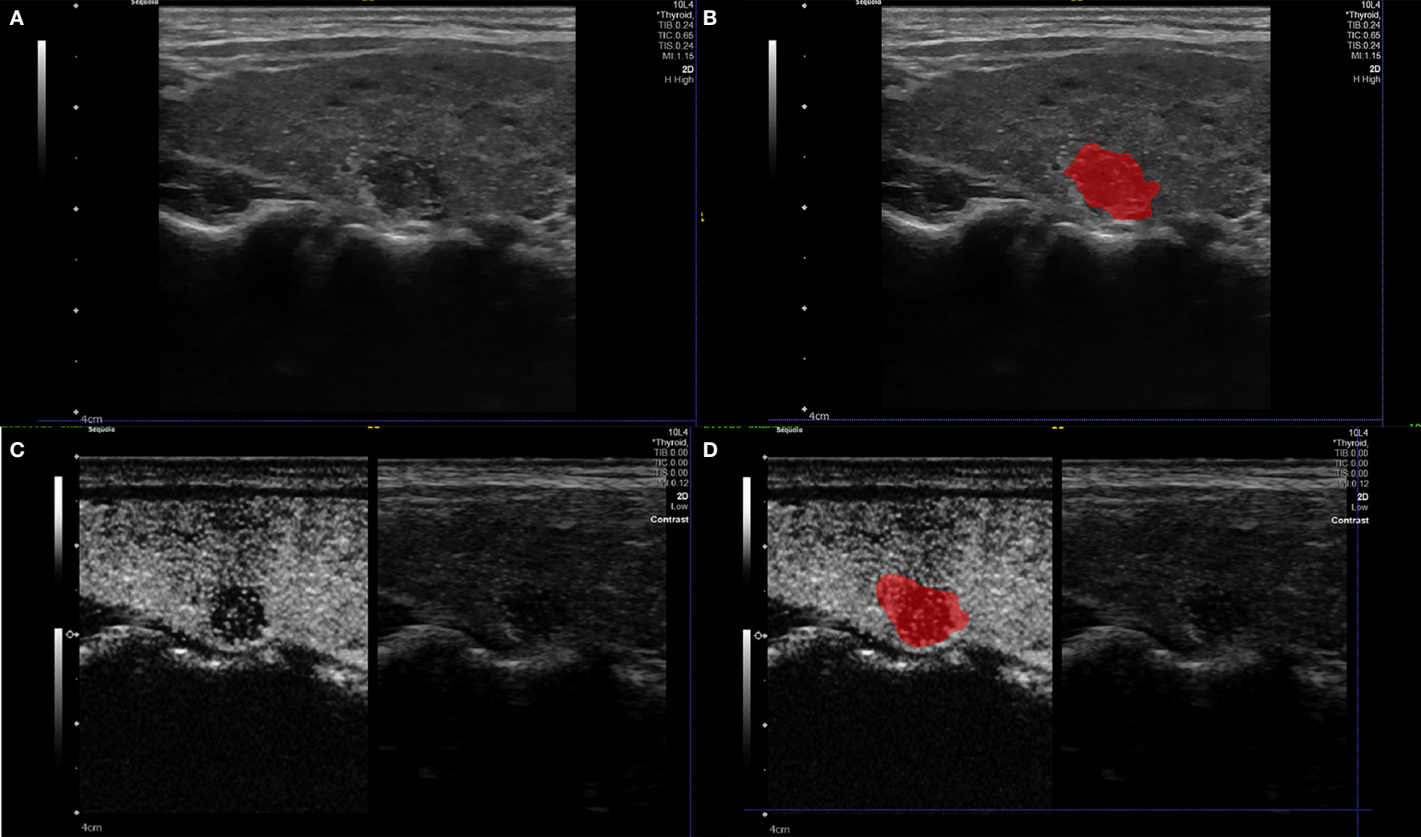
Example **(A, B)** of delineating region of interest (ROI) on a B-US image, we detailed the outline along the nodule boundary on the largest long-axis cross-section of the nodule. Example **(C, D)** of delineating region of interest (ROI) on a CEUS image. The ROI includes the target node and some of its adjacent tissues (22).

We imported the dynamic contrast-enhanced ultrasound images into the TIC software for the quantitative analysis of CEUS parameters (**Figure 2**). Based on a previous study, the differences of the quantitative parameters of CEUS between benign and malignant nodules, which suggest significant differences of peak contrast perfusion within the nodule, were found between them (13). Therefore, the single frame corresponding to the moment of peak contrast perfusion in the target nodules during contrast ultrasound was selected to represent the whole process for radiomics analysis. In the next step, we normalized the grayscale and voxels of all images. Then, the ITK-SNAP software was used to draw an outline of the area of interest (ROI) of the lesion in B-US images and CEUS images respectively (**Figure 3**), which was the target region for radiomics features extraction. The ROI was delineated by a radiologist with over 2 years of experience in diagnostic ultrasound of thyroid disease.

### Extraction and Screening of Radiomics Features

**Figure 4:**
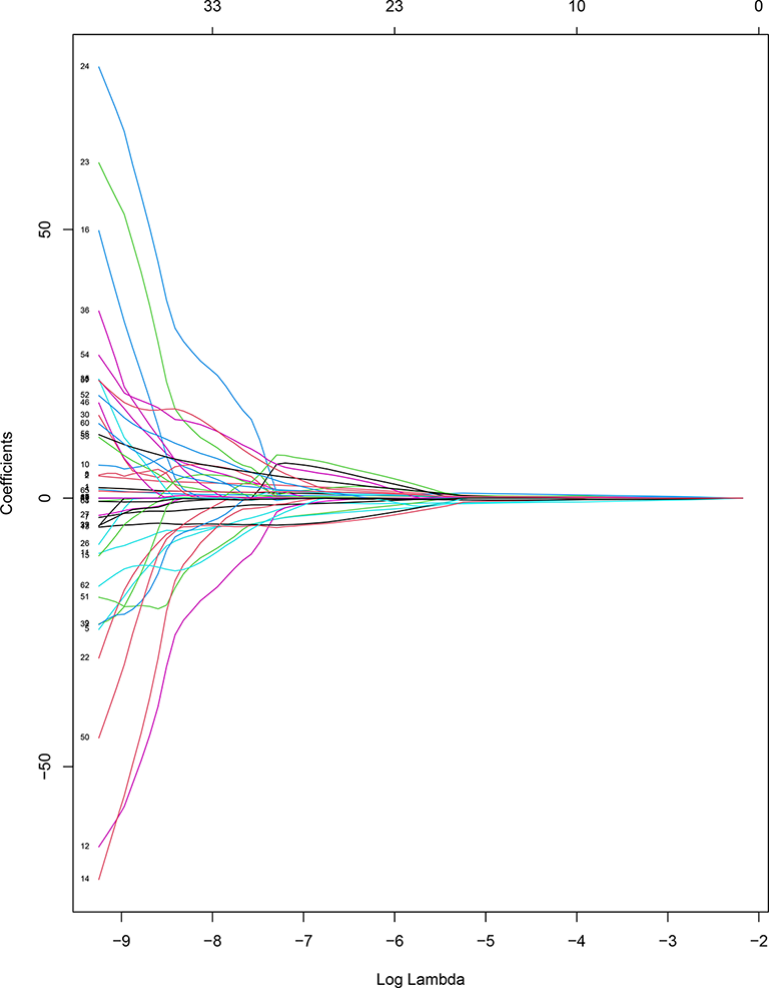
Coefficient convergence graph of B-US radiomics features.

**Figure 5:**
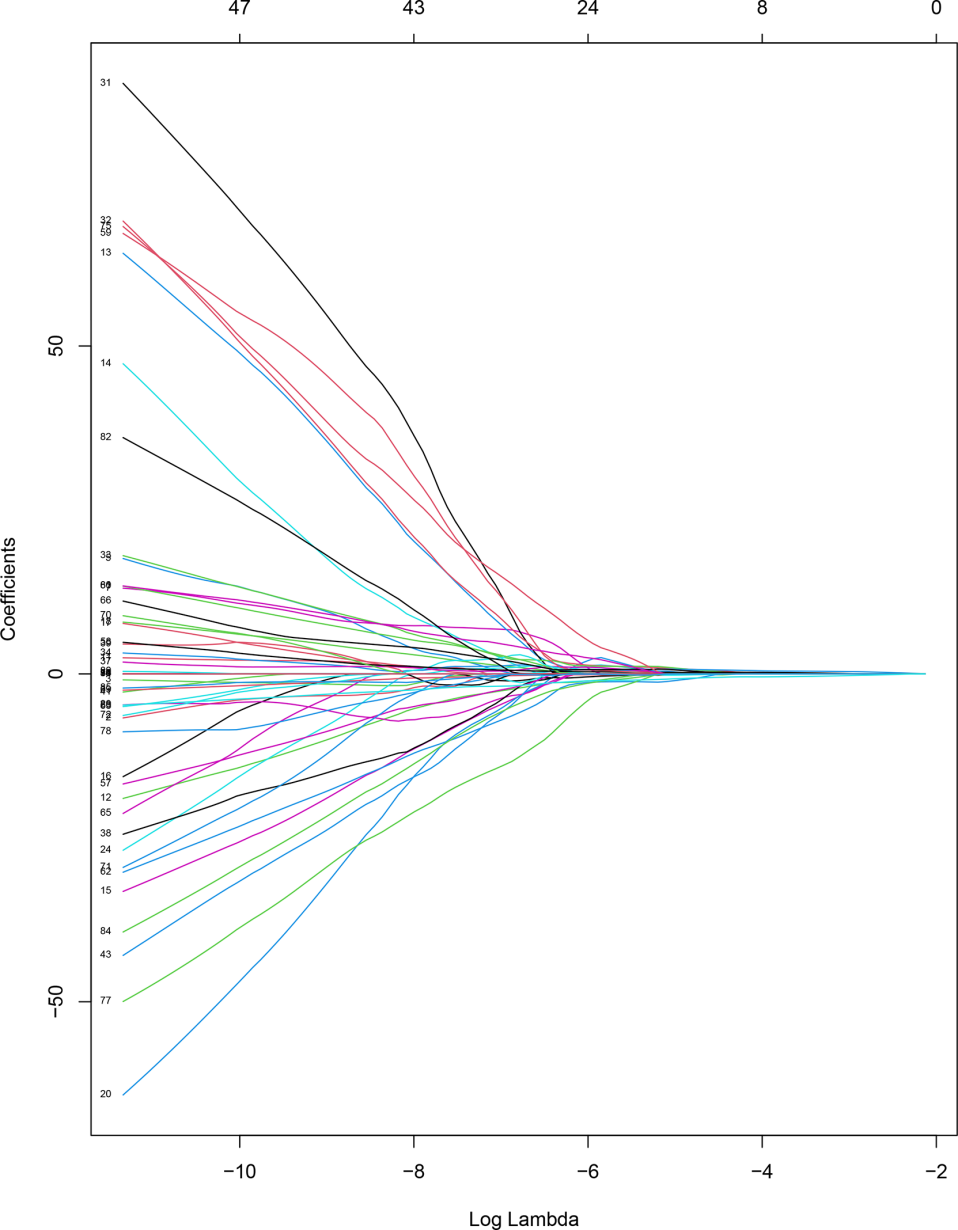
Coefficient convergence graph of CEUS radiomics features.

The task of feature extraction was completed by 3D-Slicer 4.13.0. In total, 837 radiomics features including first-order statistics, texture features, a grayscale co-occurrence matrix, grayscale tour matrix, grayscale region size matrix, domain grayscale difference matrix, and morphological features were extracted from each B-US image and single frame of CEUS images, respectively. SPSS 23.0 software was used for analyzing the normality of radiomics features. Following the results, the Kruskal-Wallis test was used for preliminary screening. Then, the patients were randomly divided into the training cohort and validation cohort in the ratio of 9:3. The least absolute shrinkage was used to reduce the dimensionality of the radiomics features in R software (**Figure 4**, **5**), we obtained the final radiomics features for logistic regression analysis to build models for predicting the nature of thyroid nodules. Last, we verified the diagnostic performance of these models in the validation cohort.

### Building and Comparison of the Models

TI-RADS + CEUS model: According to the diagnosis criteria of improved TI-RADS and combined with the pattern of contrast-enhanced ultrasound (5), a senior radiologist made risk grading diagnoses of 123 nodules for malignancy in turn. Based on the grades, logistic regression was used to build the TI-RADS + CEUS model.

Radiomics models: Based on the extracted radiomics features from different modality ultrasound images, logistic regression was used to build the B-US radiomics model and CEUS radiomics model. Later, we combined B-US radiomics features and CEUS radiomics features to build the B-US + CEUS radiomics model.

Then, we evaluated the performance of the four types of models for diagnosing the nature of thyroid nodules by comparing the area under the ROC curve.

### Statistical Analysis

The statistical analysis and plots were performed using SPSS 23.0 and MedCalc19.6.0. The continuous quantitative data are shown as the mean ± standard deviation. The categorical and rank variables are shown as the number of cases and percentages. The chi-square test, Wilcoxon test, or Fisher`s exact test were applied, as appropriate, to compare the differences between benign and malignant groups. The sensitivity and specificity, positive prediction value (PPV) and negative prediction value (NPV), accuracy, and AUC in the nature of thyroid nodule diagnoses by the B-US radiomics model, CEUS radiomics model, B-US+CEUS radiomics model, and TI-RADS+CEUS model were calculated. The AUC was used to compare the diagnostic performance of the different models. Delong’s test was used to test the difference in diagnostic performance between radiomics models and the TI-RADS+CEUS model. Results with P<0.05 meant that the difference was statistically significant.

## Results

A total of 123 nodules from 123 patients were enrolled in our study. Of which 95 nodules were excised by resection and FNAB was performed on 28 cases. The pathological types of all nodules are listed as follows: 94 malignant nodules and 29 benign nodules were included in the 123 cases of nodules. Among the benign nodules, 21 were nodular goiters, 3 were adenomas, 2 were adenomatous nodular goiters, and 3 were inflammatory lesions. Among the malignant nodules, 45 malignant nodules were papillary carcinomas, 49 malignant nodules were papillary thyroid microcarcinomas.

The results (**Table 1**) show that gender and age of the patients were not found to differ significantly between benign and malignant groups (*P*>0.05). Among the sonographic features of thyroid nodules, we found no statistical significance in location, components, and contrast enhancement pattern of nodules (*P*>0.05). However, a statistic difference in the aspect ratio was observed in the benign group compared with the malignant group (*P*<0.05). We witnessed the statistically significant difference in the echoes, border, and margin, as well as significant differences in the presence of microcalcifications inside the nodules and grades of nodules in the benign group compared with the malignant group (*P*<0.01)

**Table 1:** Comparison of the general information between the benign and malignant groups.

Parameter		Malignant group	Benign group	*P value*
Age	Average	43.734 ± 1.189	48.069 ± 2.177	0.941
Gender	Male	23 (24.47%)	4 (13.79%)	0.225
	Female	71 (75.53%)	25 (86.21%)	
Location	Left lobe	45 (47.87%)	14 (48.27%)	1.000
	Gap	7 (7.45%)	2 (6.90%)	
	Right lobe	42 (44.68%)	13 (44.83%)	
Components	Solid	88 (93.62%)	24 (82.76%)	0.156
	Cystic solid mixed	6 (6.38%)	5 (17.24%)	
Echo	Hypoechogenicity	85 (90.42%)	17 (58.62%)	<0.01
	Equalechogenicity	4 (4.26%)	4 (13.79%)	
	Hyperechogenicity	5 (5.32%)	8 (27.59%)	
Border	Well-defined	15 (15.96%)	17 (58.62%)	<0.01
	Ill-defined	79 (84.04%)	12 (41.38%)	
Margin	Regular	39 (41.49%)	22 (75.86%)	<0.01
	Irregular	55 (58.51%)	7 (24.14%)	
Tall than wide Shape	>1	41 (43.62%)	5 (17.24%)	<0.05
	<1	49 (52.13%)	24 (82.76%)	
	=1	4 (4.25%)	0 (0)	
Calcifications	Microcalcifications	64 (68.09%)	6 (20.69%)	<0.01
	No Microcalcifications	30 (31.91%)	23 (79.31%)	
Contrast enhancement pattern	Low enhancement	82 (87.23%)	20 (68.97%)	0.053
	Equal enhancement	9 (9.58%)	7 (24.14%)	
	High enhancement	3 (3.19%)	2 (6.89%)	
Grade of TI-RADS+CEUS	3	0 (0)	7 (24.14%)	<0.01
	4a	16 (17.02%)	12 (41.38%)	
	4b	36 (38.30%)	6 (20.69%)	
	5	42 (44.68%)	4 (13.79%)	

**Figure 6:**
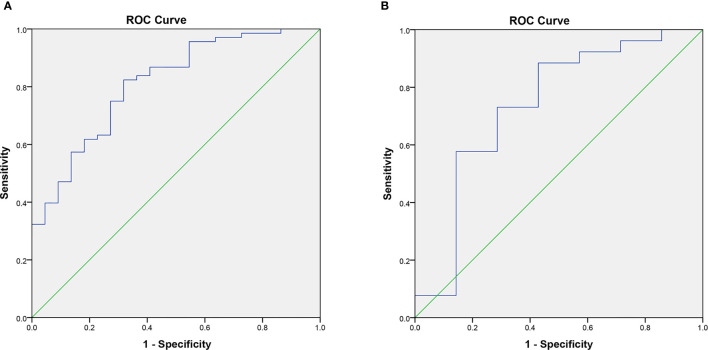
The AUC of the B-US radiomics model in the training cohort **(A)** and validation cohort **(B)**.

In the results, we obtained four radiomics features (**Table 2**) from B-US images to build the logistic regression model. For the differential diagnosis of benign and malignant thyroid nodules, the analysis of ROC curves indicated that the AUCs of the B-US radiomics model were 0.811 and 0.736 (**Figure 6**, **Table 3**) in the training cohort and the validation cohort, respectively. The results demonstrated that the four radiomics features extracted from B-US images had a good ability to distinguish the nature of thyroid nodules.

**Table 2:** The B-US radiomics features.

B-US Radiomics Feature
original_ngtdm_Strength wavelet-LLH_glszm_ZoneEntropy wavelet-LHL_glszm_GrayLevelVariance wavelet-LHH_gldm_LargeDependenceHighGrayLevelEmphasis	

**Table 3:** Comparison of information in the training and validation cohorts.

Cohort	Malignant	Benign	AUC	
			B-US Radiomics	CEUS Radiomics
Training cohort	68	22	0.811	0.770
Validation cohort	26	7	0.736	0.736

**Figure 7:**
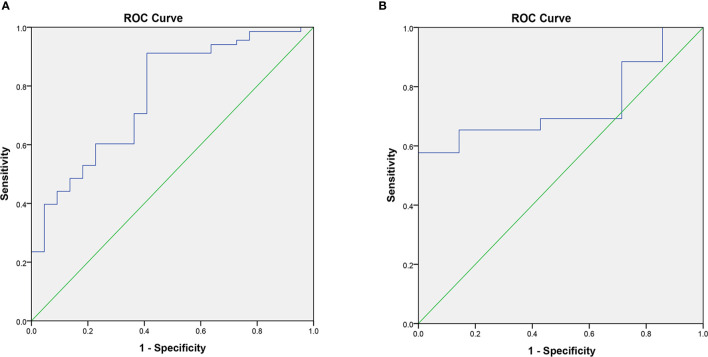
The AUC of the CEUS radiomics model in the training cohort **(A)** and validation cohort **(B)**.

Finally, we obtained six radiomics features (**Table 4**) from CEUS images to build the logistic regression model. For the differential diagnosis of benign and malignant thyroid nodules, the analysis of ROC curves suggested that the AUCs of the US radiomics model were 0.770 and 0.736 (**Figure 7**, **Table 3**) in the training cohort and the validation cohort, respectively. The results demonstrated that the six radiomics features extracted from CEUS images had a good ability to distinguish the nature of thyroid nodules.

**Table 4:** The CEUS radiomics features.

CEUS radiomics features	
wavelet-LHL_glrlm_LongRunLowGrayLevelEmphasis wavelet-HLL_glcm_MaximumProbability wavelet-HLL_glrlm_LongRunHighGrayLevelEmphasis wavelet-HLL_glrlm_RunVariance wavelet-HLH_gldm_DependenceVariance wavelet-LLL_ngtdm_Contrast

**Figure 8:**
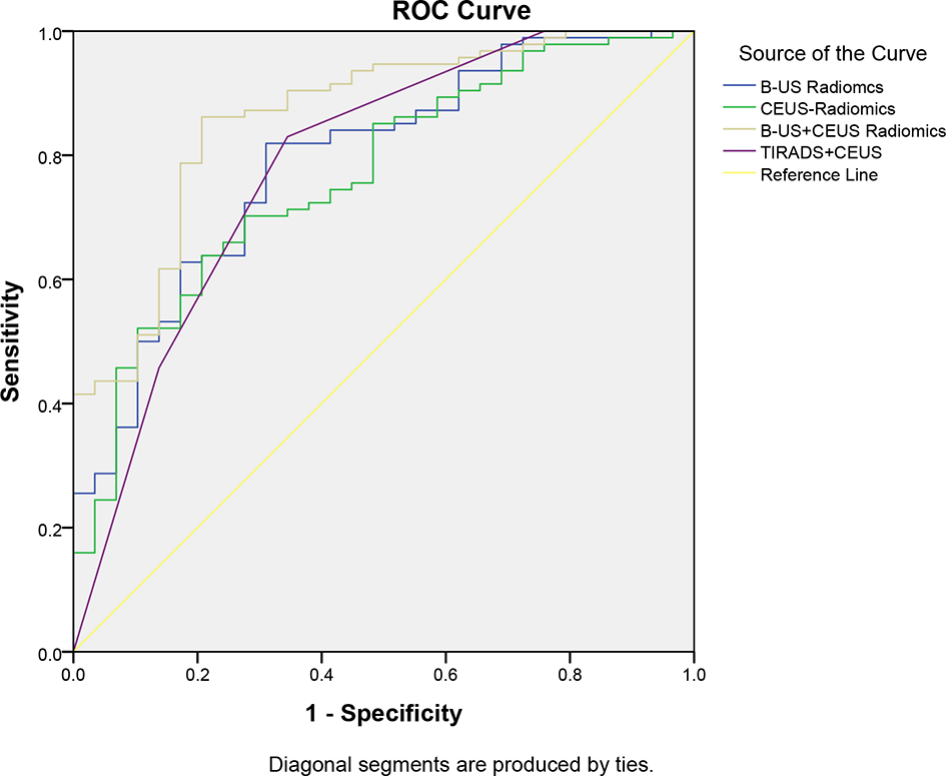
Comparison of the AUCs of the four types of models.

In our study, according to the result (**Tables 5**, **6**, **Figure 8**), we can learn that the sensitivity of the B-US radiomics model, CEUS radiomics model, B-US+CEUS radiomics model, and TI-RADS+CEUS model in the identification of benign and malignant thyroid nodules was 97.87%, 96.81%, 94.68%, and 100%, their specificity was 31.03%, 27.59%, 51.72%, and 24.14%, their accuracy was 82.11%, 80.49%, 84.55%, and 82.11%, their PPV was 82.14%, 81.25%, 86.41%, and 81.03%, their NPV was 81.82%, 72.72%, 75.00%, and 100%, and the AUCs of them were 0.791, 0.766, 0.861, and 0.785, respectively.

**Table 5:** Comparison of the diagnostic effectiveness of four types of models.

Models	Sensitivity (%)	Specificity (%)	PPV (%)	NPV (%)	Accuracy (%)
B-US radiomics	97.87	31.03	82.14	81.82	82.11
CEUS radiomics	96.81	27.59	81.25	72.72	80.49
B-US+CEUS radiomics	94.68	51.72	86.41	75.00	84.55
TI-RADS+CEUS	100	24.14	81.03	100	82.11

**Table 6:** Comparison of the AUCs of four types of models.

Models	AUC	95%CI (AUC)		*P* value	
		lower bound	upper bound	Compare to TI-RADS+CEUS	Compare to B-US+CEUS radiomics
B-US radiomics	0.791	0.699	0.883	0.935	0.013
CEUS radiomics	0.766	0.671	0.861	0.743	0.026
B-US+CEUS radiomics	0.861	0.785	0.938	0.217	–
TI-RADS+CEUS	0.785	0.681	0.889	–	0.217

Compared to the TI-RADS+CEUS model, Delong’s test showed there was no difference in the diagnostic performance between the US radiomics model, CEUS radiomics model, B-US+CEUS radiomics model, and TI-RADS+CEUS model (*P*>0.05). This indicated that the B-US model and CEUS model and B-US+CEUS radiomics model have comparable diagnostic performance to the TI-RADS+CEUS model. On the other hand, the AUC of the B-US+CEUS radiomics model was the highest among the four models. A significant difference was observed between the single B-US radiomics model or CEUS radiomics model and the B-US+CEUS radiomics model (*P*<0.05). Further analysis found that the specificity, accuracy, and PPV of the B-US+CEUS radiomics model were also the highest. However, its NPV was relatively low compared to the others.

## Discussion

The incidence of thyroid cancer has continued to grow rapidly in recent decades. According to statistics, there were 586,202 new cases of thyroid cancer worldwide in 2020, accounting for 3.0% of the total number of cancer cases and ranking 9th in incidence rate (14). Therefore, it is crucial to adopt appropriate treatment by identifying the nature of thyroid nodules accurately and effectively. After high-resolution ultrasound was combined with elastography and imaging technology, it has shown a very valuable performance in diagnosing the nature of thyroid nodules (15–17). However, the nature of certain nodules remains unclear. FNAB also suffers from inadequate sampling rate, and the invasive nature of it would lead to complications (2, 7, 18). Therefore, radiomics analysis can be an effective and promising non-invasive way for predicting the malignancy of thyroid nodules.

Recent studies from numerous researchers have reported that radiomics features extracted from B-US images can be used for the risk prediction of the malignancy in thyroid nodules (19, 20), which was also confirmed in our study, where the diagnostic performance of the B-US radiomics model was similar to the senior radiologist. Meanwhile, the radiomics studies based on multi-modality images is also gradually being developed. A study has proven that the combination of B-mode ultrasound and strain elastography ultrasound, applying radiomics analysis, could further improve the estimate accuracy of lymph node metastasis in patients with papillary thyroid carcinomas (21).

As far as we know, no study has yet determined whether the use of CEUS images for radiomics analysis can provide valuable insights for diagnosis of the nature of thyroid nodules.

Liu et al. (22) demonstrated that a deep learning-based radiomics model, designed for analyzing contrast-enhanced ultrasound, could not merely achieve an accurate preoperative prediction of the progression-free survival for radiofrequency ablation and surgical resection, but could also contribute to the optimized treatment selection between patients with very early or early-stage hepatocellular carcinomas. This demonstrates that the results of radiomics analysis in CEUS images can also provide valuable information for clinical diagnosis and treatment. In our study, we selected single-frame images corresponding to the moment of peak intra-nodal perfusion in thyroid nodules to assess the value of radiomics analysis in CEUS images for diagnosing the nature of thyroid nodules. The results showed that the CEUS radiomics model exhibited equally good diagnostic performance compared to the TI-RADS+CEUS model. Moreover, the B-US+CEUS model showed superior diagnostic performance, highlighted by a specificity of 51.72%, a significant increase compared to the others; its accuracy and PPV were also at the highest. Although the sensitivity and NPV were not very good compared to the TI-RADS+CEUS model and B-US radiomics model, it still attained the best diagnostic performance after balancing all the differences.

The result suggested that the information provided by B-US radiomics features and CEUS radiomics features may complement each other in diagnosis of the nature of thyroid nodules, both of which may have a strong correlation with certain pathological features of thyroid cancer, but this correlation has not yet been revealed. In any case, the combination of both radiomics features have presented a significant improvement in diagnostic performance that is a promising prospect for clinical application.

However, the present study also had several limitations. First of all, the subjects were recruited from a single center, and the sample size was small. Besides, in view of the particularity of this examination, most nodules were grade 4a-4b in this experiment, so these limitations increased the likelihood of bias. As a result, there was no statistical significance in components and contrast enhancement patterns of nodules between the benign and malignant groups and a reduced specificity of all models in this study. Second, due to the limitation of technology, we only selected a single frame of the CEUS images to replace the whole process of perfusion, so other factors that have not been taken into consideration could have affected the diagnostics.

In the future, multi-center, large sample data are desired to further confirm the findings of our study. We expect to search for more advanced and effective technical methods to extract the radiomics features of dynamic images, and carry out more comprehensive and multi-faceted evaluation.

## Conclusion

In summary, both the B-US radiomics features and CEUS radiomics features have the ability of qualitative diagnosis of thyroid nodules. By combining the two radiomics features we can obtain higher prediction performance, which shows a highly valuable clinical application in the diagnosis of thyroid nodules.

## Data Availability Statement

The original contributions presented in the study are included in the article/supplementary material. Further inquiries can be directed to the corresponding author.

## Ethics Statement

The studies involving human participants were reviewed and approved by Institutional Review Board of the Third Xiangya Hospital CSU. The patients/participants provided their written informed consent to participate in this study.

## Author Contributions

SG did the first draft. PZ and YZ provided instructive advice and useful suggestions for this manuscript. YFZ and LJ provided statistical advice for this manuscript. All authors contributed to the article and approved the submitted version.

## Funding

This work was supported by grants from the National Natural Science Foundation of China (No. 81871367).

## Conflict of Interest

The authors declare that the research was conducted in the absence of any commercial or financial relationships that could be construed as a potential conflict of interest.

## Publisher’s Note

All claims expressed in this article are solely those of the authors and do not necessarily represent those of their affiliated organizations, or those of the publisher, the editors and the reviewers. Any product that may be evaluated in this article, or claim that may be made by its manufacturer, is not guaranteed or endorsed by the publisher.
